# Incidence of Pediatric Cancers in French Guiana: How Does It Compare to Global Estimates?

**DOI:** 10.3390/cancers16101829

**Published:** 2024-05-10

**Authors:** Mathieu Nacher, Qiannan Wang, Lindsay Osei, Benjamin Faivre, Narcisse Elenga, Antoine Adenis, Nathalie Deschamps, Kinan Drak Alsibai

**Affiliations:** 1 Centre d’Investigation Clinique (INSERM 1424), Institut Santé des Populations en Amazonie, Centre Hospitalier de Cayenne, 97300 Cayenne, French Guiana; antoine.adenis@ch-cayenne.fr; 2 Université de Guyane, 97300 Cayenne, French Guiana; narcisse.elenga@ch-cayenne.fr; 3 Registre des Cancers de Guyane, Centre Hospitalier de Cayenne, 97300 Cayenne, French Guiana; qiannan.wang@ch-cayenne.fr (Q.W.); kdrak.alsibai@doctor.com (K.D.A.); 4 Service de Pédiatrie, Centre Hospitalier de Cayenne, 97300 Cayenne, French Guiana; lindsay.osei@ch-cayenne.fr (L.O.); benjamin.faivre@ch-cayenne.fr (B.F.); 5 Service de Neurologie, Centre Hospitalier de Cayenne, 97300 Cayenne, French Guiana; nathalie.deschamps@ch-cayenne.fr; 6 Service d’Anatomo-Pathologie, Centre Hospitalier de Cayenne, 97300 Cayenne, French Guiana; 7 Centre de Ressources Biologiques Amazonie, Centre Hospitalier de Cayenne, 97300 Cayenne, French Guiana

**Keywords:** pediatric cancers, leukemia, central nervous system tumors, sarcoma, lymphoma, French Guiana

## Abstract

**Simple Summary:**

French Guiana is a singular French territory in South America. The certified cancer registry collected data about cancers throughout French Guiana between 2003 and 2017, which allowed us to study pediatric cancers. There were 164 solid tumors or hematologic malignancies diagnosed in children under the age of 15 years. There was no significant temporal trend during the study period. The three most common causes of cancer were leukemias—mostly lymphoblastic—, CNS tumors, and sarcoma. The standardized incidence of pediatric cancers in French Guiana was similar to those in Western Europe and North America. Here we showed that, overall, contrary to our assumption and to trends in tropical countries, the incidence of pediatric cancers was in a range between Western Europe and North America. Quality cancer registry data in this tropical region confirm the suspicion that lower incidences in tropical low- and middle-income countries are likely to result from incomplete diagnosis and data collection.

**Abstract:**

French Guiana is a French territory in South America. The exposome of persons living there is quite different from that in mainland France and the ethnic make-up of the population is also quite different. Poverty is also widespread with difficulties in accessing care magnified by the low medical-professional density. In this singular context, we aimed to measure the incidence of pediatric cancers and to compare it with other continents. We used French Guiana’s certified cancer registry to study this between 2003 and 2017. Incidences were standardized using the world population with three strata: 0–4 years, 5–9 years, and 10–14 years. There were 164 solid tumors or hematologic malignancies diagnosed in children under the age of 15 (92 in boys and 72 in girls). Over the study period, the standardized incidence rate was 14.1 per 100,000 among children aged under 15 years. There was no significant trend during the study period. The three most common causes of cancer were leukemias—mostly lymphoblastic—CNS tumors, and sarcoma. The standardized incidence of pediatric cancers in French Guiana was similar to those in Western Europe and North America. As others have discovered, we found that males tended to be more likely to develop cancer, notably leukemia, CNS tumors, sarcoma, and retinoblastoma. As elsewhere, the predominant cancer types changed with age. Our initial assumption was that given the singular context of French Guiana, there may have been differences in pediatric cancer incidences. Here we showed that overall, contrary to our assumption and to trends in tropical countries, the incidence of pediatric cancers was in a range between Western Europe and North America with some apparent but non-significant differences in the main types of cancers observed in global statistics. Quality cancer registry data in this tropical region confirm the suspicion that lower incidences in tropical low- and middle-income countries are likely to result from incomplete diagnosis and data collection.

## 1. Introduction

In France, as in most high-income countries, childhood cancers are much less common than adult cancers, and their types differ from adult cancers—mainly consisting of leukemia, central nervous system tumors, lymphomas and neuroblastomas [1,2,3,4]. They often occur before the age of 5 years and represent 1 to 2% of all cancers. Nevertheless, they represent a major public-health problem since they are the leading cause of death in children after accidental causes. In comparison with adults, global quality statistics for pediatric cancers are often lacking because collection is often neglected, notably in low- and middle-income countries, and because smaller numbers are more sensitive to imprecision [1].

In French Guiana, a French territory in South America, in contrast to the aging population in mainland France, the population is multi-ethnic and young [5,6] and the epidemiological situation of childhood cancers is poorly known. Generally speaking, however, French Guiana has many specific epidemiological features in terms of incidences of certain cancers compared to mainland France [7]. Overall, standardized cancer incidence among adults is lower than in France but some cancers have higher incidences—often infection-related cancers such as cervical or gastric cancer [8,9].

The exposome of persons living in French Guiana is quite different from that of mainland France [10]. The ecosystem of pathogens and environmental exposures—some of which may be carcinogenic [11,12]—are very different from those in mainland France. In addition, the region is faced with significant social inequalities in health, which hamper access to care for patients [13,14]. Over half the population lives below the national poverty line [15]. Furthermore, the lack of specialists often means that patients have to be evacuated to mainland France to be treated, according to the French National Cancer Institute (INCA) standards. In French Guiana, three out of five births are to foreign mothers. These mothers are often poor and have little knowledge of the healthcare system, which can lead to delays in specialized care [16]. They often suffer from food insecurity [17] and heavy metal intoxication [18], which affect the fetus from conception. A high prevalence of abnormal heavy metal concentrations results from a combination of geological factors and human factors (mercury from goldmining, lead from cooking or hunting).

Although environmental factors may not be as important as in adult cancers, the prevalence of these adverse conditions in pregnancy and childhood is worrying. Studies in the USA on pediatric cancers suggested there were differences in different cancer susceptibilities between different ethnicities [1]. The population of French Guiana originates from very different ancestral populations, which may affect cancer incidences [19]. In this overall context, we hypothesized that pediatric cancer incidences may be different from Europe (French Guiana is an ultraperipheral region of Europe) and from South America (French Guiana is in South America). To the best of our knowledge, few data exist from the Amazon basin countries to describe childhood cancers [20,21]. French Guiana is a rich Amazonian territory, where a significant proportion of the population is poor, but mostly benefits (87%) from a universal French health system. In this context, there is an opportunity to specify the epidemiology of childhood cancers, with quality data despite the widespread poverty, which could be useful for countries in the region with lower health resources.

In order to clarify this epidemiological situation, the French Guiana Cancer Registry carried out a retrospective descriptive study of the incidence of different types of childhood cancer. This study used the registry database, which includes all new cases of cancer diagnosed in Guianese residents, regardless of tumor location and therapeutic management. The results obtained are based on the analysis of data concerning all incident cancers occurring in children under 15 years of age—pediatric cases, residing in French Guiana at the time of diagnosis, over the period from 2003 to 2017. After describing the situation in French Guiana, we compared our standardized results with global standardized estimates.

## 2. Materials and Methods

### 2.1. Background and Population

French Guiana is France’s largest administrative “département”, covering an area of almost 84,000 km^2^. It is located in South America, between Suriname and Brazil, and over 90% of its surface area is covered by the Amazon rainforest.

Between 2003 and 2017, its population rose from 184,792 to 268,700, representing a growth of 3.2% per year. The under-15 population grew from 66,133 to 87,915 children (+2.4% per year), representing around a third of French Guiana’s total population. This makes French Guiana the fastest-growing region in France, with a low population density (three inhabitants per km^2^) and a very young population, due to a greater fertility rate than the national average (3.53 children per woman versus 1.8).

French Guiana is also distinguished by the multi-ethnic nature of its population, with Amerindian, Creole, African, Asian, and European origins, and by the diversity of languages spoken. In 2011, foreigners, mainly from Suriname, Haiti, and Brazil, accounted for 35% of the total Guianese population.

The territory faces major socio-economic inequalities. More than half the population (53%) lived below the poverty line in 2017, compared with 14% in mainland France. The average declared net income per tax household in euros is half that of mainland France. In addition, the proportion of the population covered by the Revenu de Solidarité Active (RSA) and the Couverture Maladie Universelle Complémentaire (CMU-C) is much higher than in mainland France.

The universal healthcare system, which is made up of a hospital and private healthcare system supplemented by healthcare centers (Centres Délocalisés de Prévention et de Soins, CDPS), faces a number of challenges when it comes to implementing a local and varied healthcare offering. The severe shortage of healthcare professionals, combined with incompletely diversified specialized services, complicates access to care for patients. As a result, patients with malignant tumors are most often evacuated to French Guiana for diagnosis and/or initial treatment of their cancer, prior to follow-up treatment.

Since 2005, the department has had a General Cancer Registry, which covers the entire population and records continuously and exhaustively all new cases of invasive, in situ, benign or unpredictably progressive cancers of the bladder and central nervous system (CNS), occurring since 1 January 2003 in people residing in French Guiana at the time of diagnosis, regardless of cancer location or place of care.

### 2.2. Inclusion Criteria

All malignant and benign CNS tumors diagnosed in children aged under 15 years during the study period from 1 January 2003 to 31 December 2017 (date of last validated collection year) were included in this study.

### 2.3. Exclusion Criteria

Benign tumors (excluding the CNS), basal cell carcinomas, recurrences, metastases and malignant tumors in children living outside the territory at the time of diagnosis were excluded.

### 2.4. Collected Data

Data collected for each tumor included diagnosis date, birth date, gender, histological classification, and tumor location (topography). We used MySQL 8.0 and MOVIBASE for data collection. The data presented were analyzed in terms of distribution by gender, five-year age group and cancer type.

### 2.5. Population Data

Population data were obtained from censuses carried out by the Institut National de la Statistique et des Etudes Economiques (INSEE), which provide an estimate of the French Guianese population by five-year age group. They were used to calculate standardized incidences of childhood cancer.

### 2.6. Judgement Criteria

To study the distribution and incidence of the different types of childhood cancer in French Guiana, the following indicators were calculated in total, by sex and by type of cancer, over the entire 2003–2017 period: The number of tumors, sex ratio, direct age-standardized incidence rates per 100,000 person-years (using the world population [22]) in order to allow comparisons between different world regions.

### 2.7. Comparative Data

Standardized incidence rates for French Guiana were compared with those found in the scientific literature for different regions of the world. We computed chi2 tests using the number of the most frequent cancers (leukemia, CNS, lymphoma, sarcoma) and the number of person-years in French Guiana with global figures extracted from the review in [1]. In order to determine if the annual incidence of pediatric cancers significantly varied, we used the free software for Joinpoint regression (version 5.1.0) [23].

### 2.8. Ethical and Regulatory Aspects

The Cancer Registry is a certified registry affiliated with the Francim network of cancer registries [24]. The national context for registries has recently evolved with new regulations, notably the General Data Protection Regulation (GDPR) requiring an adaptation of the frames of reference compatible with their type of activity [25]. By the end of 2022, following advice from the CNIL and the Health Data Hub (HDH), we overhauled the data protection impact assessment (PIA) of the Registry (data security procedures, in particular), and worked with the CNIL to adapt to the new regulations.

## 3. Results

From 2003 to 2017, the French Guiana Cancer Registry recorded 6709 new cases of cancer, all sexes and ages combined. Of these, 164 were diagnosed in children under the age of 15 (92 in boys and 72 in girls), an average of 10.9 cases per year. These pediatric tumors accounted for 2.4% of total cancers (2.5% and 2.4%, respectively, of all cancers in men and women).

Over the study period, standardized incidence rates were estimated at 8 cases per 100,000 boys under 15 and 6.1 cases per 100,000 girls under 15.

**Figure 1:**
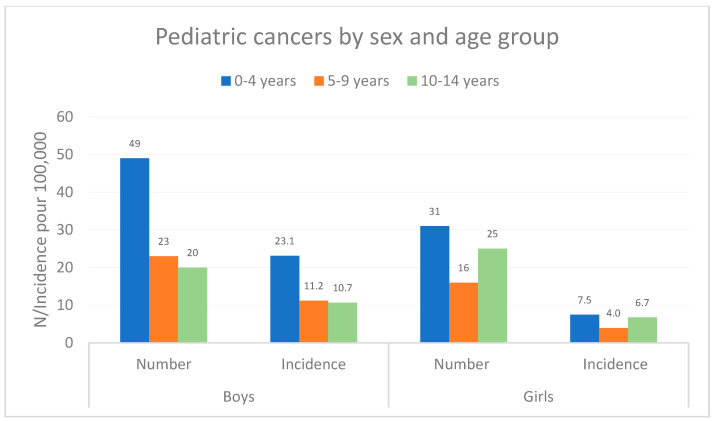
Distribution of the number and incidence of pediatric cancers by age group and sex in French Guiana, 2003–2017.

The distribution of the number of pediatric tumors and standardized incidence by age group and gender is shown in Figure 1. The majority of cancers were observed before the age of 5, in both sexes: incidence peaked at 0–4 years, then fell between 0–4 and 5–9 years. In boys, this decline was observed between ages 5–9 and 10–14 years, while in girls, incidence tended to increase again among the 10–14-year age group.

Table 1 shows the main tumors, the sex ratio, and the standardized incidence over the study period. Between 2013 and 2017, just over a third of childhood cancers of all ages and sexes were hematological malignancies, affecting mostly boys (sex ratio M/F > 1).

Among all childhood tumors (N = 164), leukemia topped the list of the two main tumor localizations, accounting for a quarter of these cancers (25.6%), followed by central nervous system tumors (23.2%). Other tumors were less represented (<10% cases per tumor site).

The median age at diagnosis was generally less than or equal to 5 years, with the exception of lymphomas (11 years) and ovarian cancers (9.5 years).

**Figure 2:**
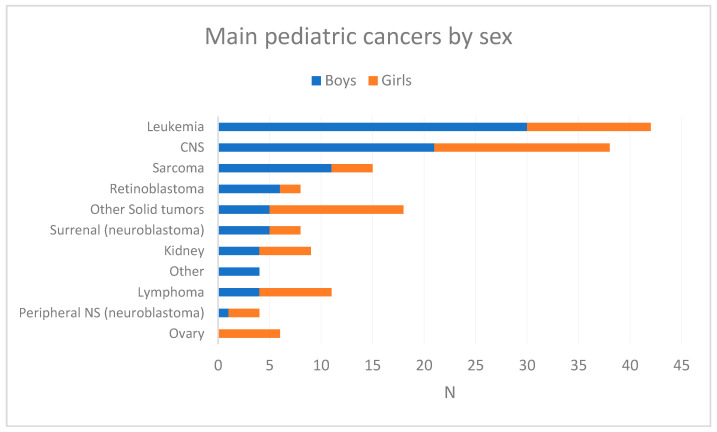
Breakdown of the main types of pediatric cancer by gender in French Guiana, 2003–2017.

Figure 2 shows the breakdown of pediatric cancers by gender. The incidences of the main pediatric tumors were higher in boys than in girls, except for lymphomas, kidney tumors, and peripheral nerve/SNA tumors.

**Figure 3:**
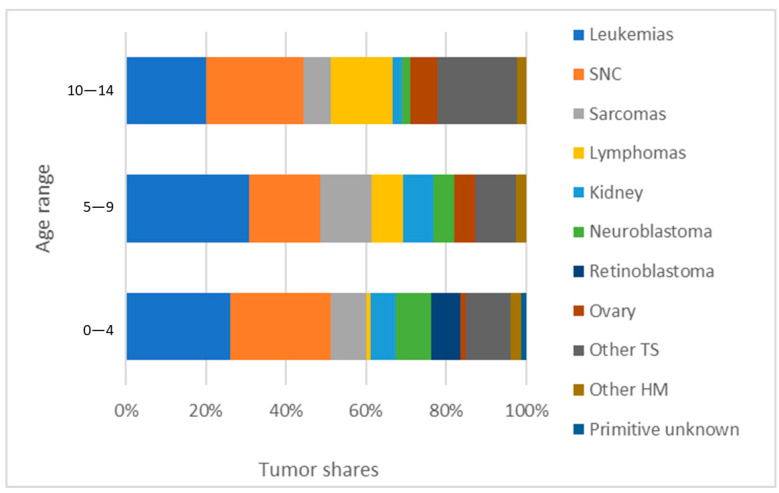
Distribution of the proportion of pediatric tumors by age in French Guiana, 2013–2017.

Figure 3 shows the distribution of the proportion of major childhood tumors by age. The proportion of leukemias was highest among 5–9-year-olds and lowest among 10–14-year-olds. The proportion of CNS tumors was minimal in the 5–9 age group, and almost equivalent in the 0–4 and 10–14 age groups.

**Figure 4:**
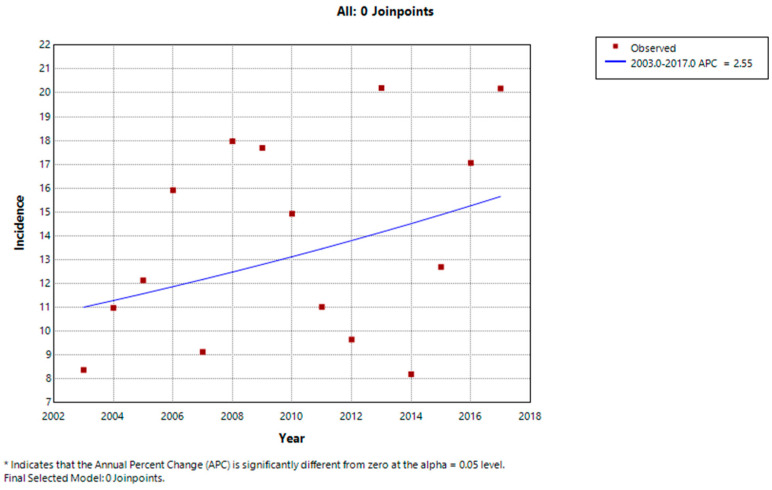
Evolution of the incidence of pediatric cancers in French Guiana, 2003–2017.

Figure 4 shows the evolution of the yearly standardized incidence of pediatric tumors between 2003 and 2017. Despite the significant fluctuations linked to the small number of cases, the comparison of standardized incidence rates by sex and year seemed to increase between 2003 and 2017, but Joinpoint regression did not find any significant increase (annual percentage change = 2.55 (−3.18 to +8.53), *p* = 0.35). No Joinpoints were detected in the period.

**Figure 5:**
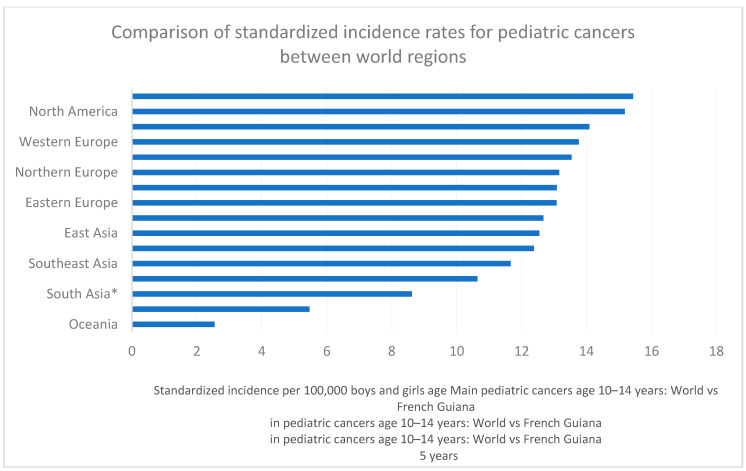
Comparison of standardized incidence rates for pediatric cancers between world regions (* India).

Figure 5 compares the standardized incidence rate for pediatric cancers in French Guiana relative to other world regions and shows that the standardized incidence in French Guiana lies between that of Western Europe and North America.

**Figure 6:**
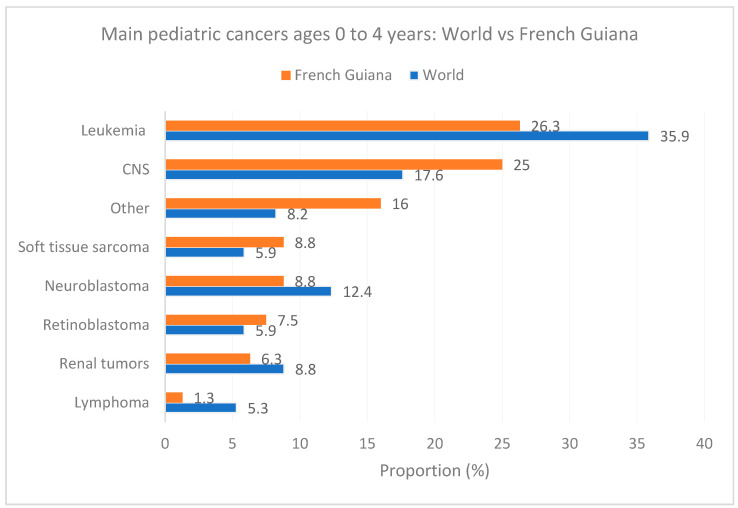
Main pediatric cancers ages 0 to 4 years: World vs. French Guiana.

**Figure 7:**
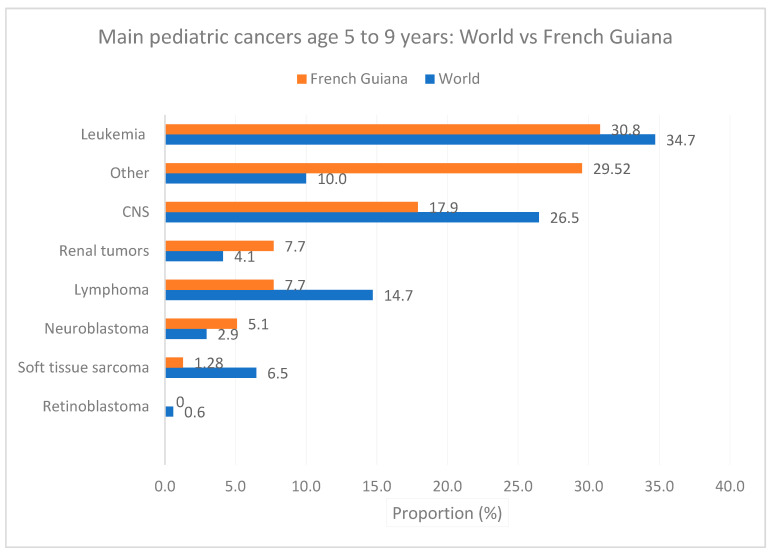
Main pediatric cancers age 5 to 9 years: World vs. French Guiana.

**Figure 8:**
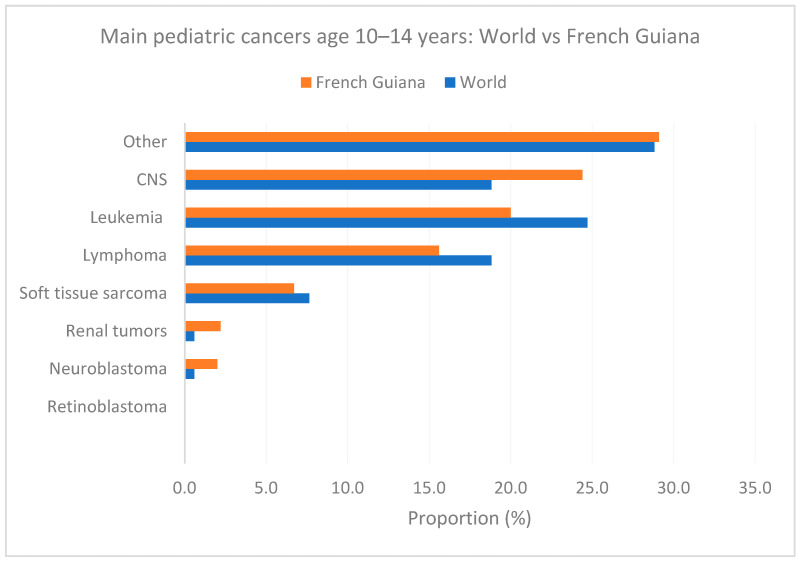
Main pediatric cancers age 10–14 years: World vs. French Guiana.

Figure 6, Figure 7 and Figure 8 show the main cancers for French Guiana and the world for ages zero to 4 years, 5 to 9, and 10 to 14 years, respectively. Although there seemed to be some differences (for instance, fewer leukemia and more CNS tumors), none of the comparisons was statistically significant (overall homogeneity chi2 *p* = 0.32 with *p*-values for specific cancers ranging from 0.7 to 0.34).

Figure 7 shows the main cancers for French Guiana and the world for ages 5 to 9 years.

Figure 8 shows the main cancers for French Guiana and the world for ages 10 to 14 years.

## 4. Discussion

Here we show that the observed standardized incidence of pediatric cancers in French Guiana was similar to that in Western Europe and North America. The three most common causes of cancer were leukemias—mostly lymphoblastic—CNS tumors, and sarcoma. Although the prevalence of HIV has been greater than 1% for over 3 decades with occasional residual vertical transmission, most infected children are under antiretroviral treatment and do not develop AIDS-defining conditions [26]; hence, lymphoma incidence was at 0.9 per 100,000 vs. 1.52 per 100,000 globally [1].

As others discovered, we found that males tended to be more likely to develop cancer, notably leukemia, CNS tumors, sarcoma, and retinoblastoma. As elsewhere, the predominant cancer types changed with age [1].

Although pediatric cancers in French Guiana are generally medically evacuated to mainland France for explorations and diagnosis, we do not anticipate any loss of information. Indeed, the Cancer Registry continuously and exhaustively records new cases of cancer diagnosed from 1 January 2003 onwards, corresponding to invasive and/or in situ tumors in patients residing in French Guiana, whatever the cancer location and place of care (whether patients are diagnosed and treated in French Guiana or elsewhere). Hence, the Francim network of registries allows us to standardize data collection and centralizes all data, which enables us to complete the French Guiana database with patients from mainland France or other overseas territories. In French Guiana, as elsewhere in France, all patients must be discussed in a multidisciplinary meeting to decide on a treatment plan which makes them easy to identify. Given all this, and given the 15-year observation period which allows us to reduce fluctuations, the incidence seems to be reliable. The relatively high incidence levels for a tropical territory may thus reflect the fact that the registry thoroughly reports pediatric cancer cases. Hence, the systematic review of registries generally found a lower pediatric cancer incidence in low-income tropical countries. A frequent explanation for this is that the capacity to diagnose and the quality of reporting and data collection systems are poor and thus the incidences are underestimated. Here, the health system is the same as in France and will generally use all means to perform diagnoses and treat all patients, and data collection of the registry is standardized. Furthermore, in this sparsely populated territory an exhaustive knowledge of all individual cases is easily achievable, so overlooking cases is unlikely.

Some authors have suggested a lower incidence of leukemia in children of African ancestry, the most common one in French Guiana. Hence, the 3.5 per 100,000 incidence in French Guiana is lower than the 4.6 per 100,000 global standardized incidence rate. It has also been suspected that massive pesticide usage in Southeast Asia has contributed to the very high incidence of leukemia there [27,28,29]. The greater incidence of pediatric CNS cancer incidence in highincome countries is assumed to partly reflect difficulties in securing medical imagery in low- and middle-income countries. Here, the 3.2 per 100,000 incidence seemed greater than the 2.8 per 100,000 incidence worldwide. Overall, the incidence of pediatric cancers in tropical French Guiana is akin to that of Western Europe or North America, suggesting that when health resources are present and quality data collection is implemented, there is no great difference despite tropical environments and ethnic variations. Given the scarcity of data on pediatric cancers in the Amazonian region, the present results will potentially be useful for the Amazon and Guiana shield.

The present study nevertheless has limitations. The small population leads to wide yearly fluctuations in incidence rates, further compounded by stratification by sex. However, as described above, the 15-year period and the good exhaustivity of diagnoses and data collection allow us to mitigate these problems. Socio economic and cultural factors are commonly associated with renouncing care in French Guiana [30,31] and thus, theoretically, it is possible to argue that some children may not be counted. However, cancer leads to severe illness and families eventually consult and agree to medical evacuations for treatment. Hence, in over 20 years there was only a single instance of a patient refusing medical evacuation to mainland France for treatment and even then the diagnosis was made and thus the patient was included in the statistics. Finally, given the ethnic diversity of French Guiana the question of differences in prevalences would seem an important way to identify vulnerabilities, but ethnicity is sensitive information in France and we could not study this.

## 5. Conclusions

In conclusion, our initial assumption was that, given the tropical context, widespread poverty and food insecurity, and the singular ethnic fabric of the population, there may have been differences in pediatric cancer incidences. Here we showed that, overall, contrary to our assumption and to trends in tropical countries, the incidence of pediatric cancers was in a range between Western Europe and North America with some apparent but non-significant differences in the main types of cancers observed in global statistics.

## Figures and Tables

**Table 1:** Distribution of main types of pediatric cancers, sex ratio, incidence in French Guiana, 2003–2017.

Type of Malignancy	Number	(%)	Sex Ratio	Incidence Rate *	Median Age at Diagnosis
* **MALIGNANT HEMOPATHIES** *	***57***	***34.8***	***2.0***	***4.9***	
**Leukemia**	42	25.6	2.5	3.6	4.5
*Leukemia/Precursor cell lymphoblastic lymphoma*	29	17.7	1.9	2.5	5
*Acute myeloid leukemia (AML)*	9	5.5	8.0	0.8	3
*Other leukemias*	4	2.4	3.0	0.3	-
**Lymphomas**	11	6.7	0.6	0.9	11
*Hodgkin’s lymphoma*	9	5.5	0.5	0.8	12
*Non-H. malignant lymphoma*	2	1.2	1.0	0.2	-
***Other hematological malignancies (n < 4 per location)***	4	2.4	-	0.3	-
***SOLID TUMORS***	***106***	***64.6***	***1.0***	***9.1***	
*Central Nervous System*	38	23.2	1.2	3.3	4
*Sarcomas*	15	9.1	2.8	1.3	5
*Kidney*	9	5.5	0.8	0.8	4
*Nephroblastoma*	6	3.7	0.5	0.5	-
*Adrenal gland*	8	4.9	1.7	0.7	3
*Neuroblastoma*	7	4.3	2.5	0.6	-
*Peripheral nerves and autonomic nervous system*	4	2.4	0.3	0.6	2
*Neuroblastoma*	3	1.8	0.5		-
*Eye and appendages*	8	4.9	3.0	0.3	1
*Retinoblastoma*	6	3.7	2.0	0.7	-
*Ovary*	6	3.7	-	0.5	9.5
*Other solid tumors (ST)* *(n < 4 per location)*	18	11.0	0.4	0.5	-
*Unknown primitive*	1	0.6	-	0.6	-
**TOTAL**	***164***	***100.0***	***1.3***	***14.1***	**5**

* per 100,000 children under 15.

## Data Availability

Upon reasonable request and after approval from the Commission Nationale Informatique et Libertés, data may be shared.
